# Evolution along the parasitism-mutualism continuum determines the genetic repertoire of prophages

**DOI:** 10.1371/journal.pcbi.1008482

**Published:** 2020-12-04

**Authors:** Amjad Khan, Alita R. Burmeister, Lindi M. Wahl

**Affiliations:** 1 Department of Applied Mathematics, Western University, London, Ontario, Canada; 2 Department of Ecology and Evolution, Yale University, New Haven, Connecticut, USA; 3 BEACON Center for the Study of Evolution in Action, East Lansing, Michigan, USA; Helmholtz-Zentrum fur Infektionsforschung GmbH, GERMANY

## Abstract

Integrated into their bacterial hosts’ genomes, prophage sequences exhibit a wide diversity of length and gene content, from highly degraded cryptic sequences to intact, functional prophages that retain a full complement of lytic-function genes. We apply three approaches—bioinformatics, analytical modelling and computational simulation—to understand the diverse gene content of prophages. In the bioinformatics work, we examine the distributions of over 50,000 annotated prophage genes identified in 1384 prophage sequences, comparing the gene repertoires of intact and incomplete prophages. These data indicate that genes involved in the replication, packaging, and release of phage particles have been preferentially lost in incomplete prophages, while tail fiber, transposase and integrase genes are significantly enriched. Consistent with these results, our mathematical and computational approaches predict that genes involved in phage lytic function are preferentially lost, resulting in shorter prophages that often retain genes that benefit the host. Informed by these models, we offer novel hypotheses for the enrichment of integrase and transposase genes in cryptic prophages. Overall, we demonstrate that functional and cryptic prophages represent a diversity of genetic sequences that evolve along a parasitism-mutualism continuum.

## Author summary

Prophages exhibit tremendous variation in both length and gene content, but little is understood about the gene repertoires of intact (functional) or degraded (cryptic) prophage sequences. By comparing large datasets of annotated prophage genes, we offer evidence for both the enrichment and loss of genes of specific function, resulting in various “signatures” of prophage gene content. We then use analytical and computational models to understand the evolutionary forces underlying those genomic signatures. In particular, our data analysis demonstrates that tail fiber, integrase and transposase genes are enriched in cryptic prophages. Informed by our simulation studies, we offer novel hypotheses to explain these unexpected results.

## Introduction

Bacteriophages (‘phages’), viruses that infect bacteria, are the most prevalent life form on the planet, vastly outnumbering both their bacterial hosts and all other life forms combined [1–3]. Many phages alternate between lytic and temperate life cycles. During lytic reproduction, phage replication causes host death. During temperate reproduction, the phage genome (now termed a “prophage”) quiescently integrates into the bacterial genome, leaving the host largely unharmed (assuming prophage integration did not inactivate beneficial host genes) and potentially contributing beneficial genes to the host cell. These two life cycles represent the extremes of parasitism and mutualism within a single phage genotype, but these life cycles can also evolve along a phage lineage.

While integrated in the bacterial genome, prophage sequences are subject to selection, mutation, and horizontal gene transfer (HGT). A wealth of recent evidence argues for the role of positive selection in the maintenance of prophages, which confer benefits such as immunity against other infecting phages, antibiotic resistance, resistance to environmental stress and numerous virulence factors [4]. Mutation in bacterial genomes is biased toward deletion [5–7], and thus prophage sequences are subject to mutational degradation over time. In addition, some families of prophages carry transposase genes, enabling replicative (copy-and-paste) transposition of the prophage sequence to other locations in the bacterial genome.

If a prophage retains the functional genes required for replication, nucleic acid packaging, and cellular lysis, upon induction it can resume its role as a lethal pathogen, killing the host cell while producing progeny phage particles. Spontaneous induction rates are in the range of 10^−5^ to 10^−3^ per bacterial generation [8, 9], but can be substantially increased if the bacterial host cell is in stress [10].

Genome sequencing and comparative genomics have recently revealed that prophages are far more numerous and more widely-shared across bacterial genomes than previously recognized [11, 12]. In addition, four recent studies have independently reported that the distribution of prophage lengths is bimodal [13–16], a phenomenon that may be explained by the balance between selection for mutualist prophage maintenance (via beneficial effects of prophage genes) and selection against parasitic prophage (via harmful effects of induction and cell lysis) [17].

These fundamental evolutionary forces will differentially affect prophage genes of different function. For example, short deletions might affect all prophage genes equally. Meanwhile, positive selection will affect only those prophage genes that confer a benefit to their host, and negative selection will act against only prophages that carry functional induction genes. Together, a combination of deletion and selection events will result in a distribution of prophages that vary in genome integrity and length compared to the ancestor prophage genome. These ‘degraded’ or cryptic phages may then also carry different genetic repertoires as signatures of the evolutionary forces in play. Indeed, tail fiber and integrase coding sequences were found to be significantly enriched in small prophages (see S1 Fig in [13]), but little else is understood about the gene repertoires of intact or degraded (incomplete) prophage sequences.

In this contribution, we examine the distributions of prophage genes identified in publicly available genome sequences, comparing the gene repertoires of intact and incomplete prophages. To better understand the results of this bioinformatic analysis, we also develop an analytical model and a computational (agent-based) simulation to predict the fates of distinct gene classes in prophages as they evolve along the parasitism-mutualism continuum. Our results support the roles of both positive and negative selection in the evolution and maintenance of prophage sequences with diverse genetic repertoires, and they offer explanations for both the enrichment and loss of specific gene functions in cryptic prophages.

## Methods

### Gene repertoire of sequenced prophages

We investigated bacterial genomes studied in two previously published data sets [13, 16], using the PHASTER interface [18] for rapid prophage identification and gene annotation. Data Set 1, originally studied by Bobay et al. [13], includes 624 prophages from 85 enterobacterial genomes; these sequences contain 24,877 prophage genes. Data Set 2, as studied by Leplae et al. [16], includes 760 prophages from 306 phylogenetically diverse bacterial genomes, with 28,479 prophage genes. Thus Data Set 2 presumably includes a wider diversity of prophages.

Each bacterial genome was submitted for analysis to the PHASTER web tool. PHASTER identifies prophages in the bacterial genome and classifies them as “intact”, “questionable” or “incomplete”. In addition, for each coding sequence within a putative prophage, PHASTER provides an output of gene annotations identified as BLAST hits for that sequence. We recorded all annotations for all coding sequences within prophages, and then searched these annotations for the keywords shown in Table 1 (see S1 Appendix; full details regarding the classification of prophage sequences and BLAST hits in PHASTER are provided in [18]). For the 13 phage gene classes listed in Table 1, we counted the number of prophages identified as containing at least one gene of that class, for a total of 7,150 instances of these phage gene functions occurring within a prophage. We further partitioned these data based on whether the prophage sequence was classified as “intact”, “questionable” or “incomplete” by the PHASTER algorithm.

**Table 1:** The genetic repertoire of prophages in Data Set 1 and Data Set 2.

	Number of prophages containing a gene of this type					
Gene	Count in Data Set 1			Count in Data Set 2		
	Intact	Questionable	Incomplete	Intact	Questionable	Incomplete
terminase	317	25	14	292	53	58
portal	277	9	3	283	67	48
head	299	16	25	281	79	86
injection	14	0	2	4	0	0
tail	413	46	86	419	116	141
protease	82	3	5	72	12	22
transposase	195	32	75	190	173	144
integrase	346	54	85	312	94	165
lysis	226	19	11	52	6	6
plate	121	5	0	143	20	28
capsid	225	14	18	233	50	40
lysin	235	17	19	165	32	22
flippase	2	0	20	0	1	4
**Total**	2752	240	363	2446	603	764

**Fig 1:**
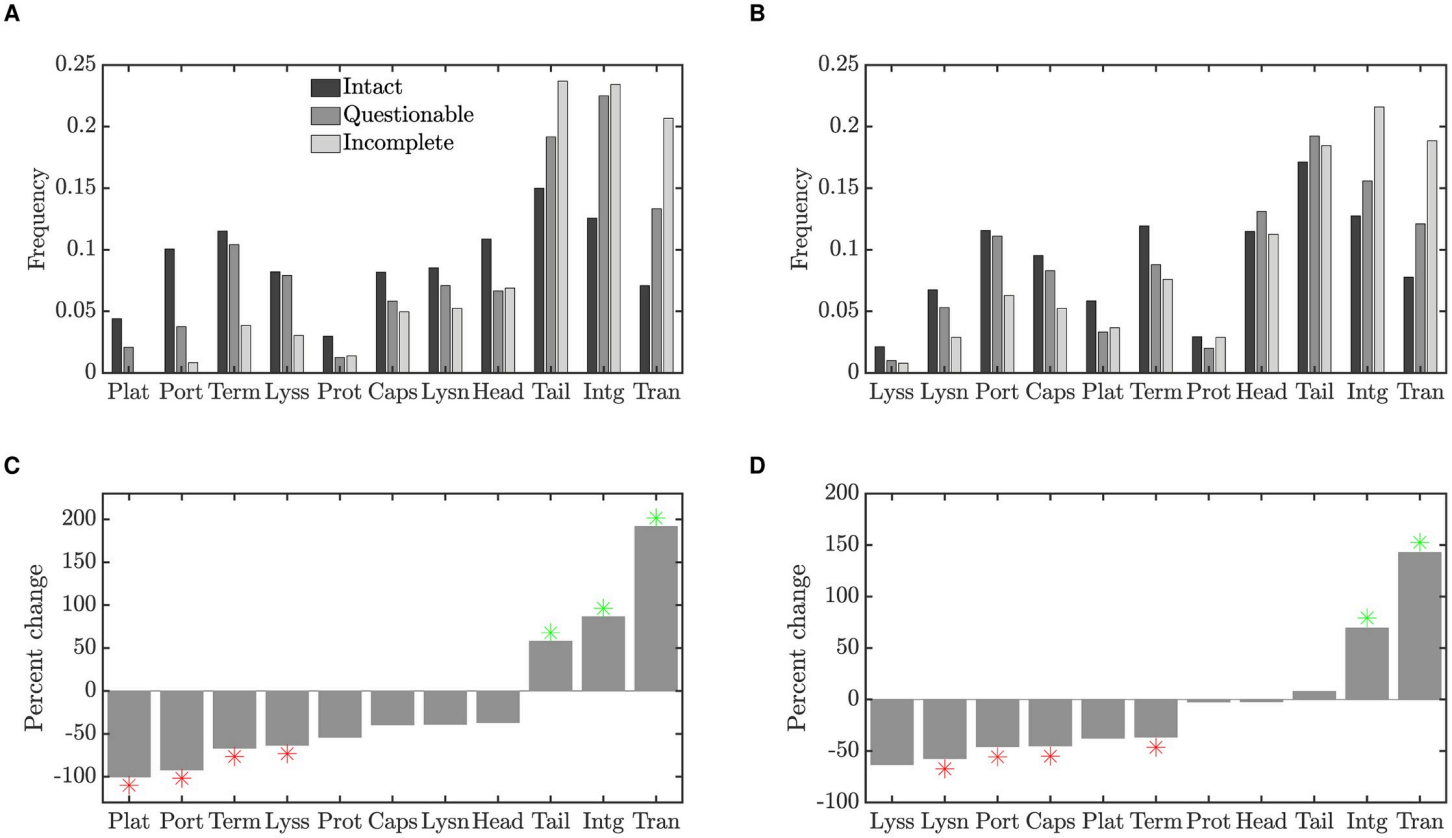
Changes in prophage gene frequencies, for intact, questionable and incomplete prophages. (A) The frequency of each gene class identified in Table 1 in prophages from Data Set 1 [13], for prophages identified as intact, questionable or incomplete. Gene classes that constituted less than 1% of the data have been excluded. Gene classes are ordered by the percent change in frequency (degree to which they are enriched in incomplete prophages, see panel C). (B) Gene frequencies as in panel A, but for Data Set 2 [16]. (C) Percent change in gene frequency in Data Set 1; the frequency of each gene class in incomplete prophages is compared to the baseline frequency of that class in intact prophages. Frequencies that were significantly lower (red) or higher (green) than expected by chance with a two-sided 5% significance threshold are indicated by stars. Thus red stars indicate gene classes that are preferentially lost, while green stars indicate classes that are enriched in short prophages. (D) Percent change in gene frequency for Data Set 2.

Fig 1 plots the frequency of each gene class in intact (*f*_int_), questionable and incomplete (*f*_inc_) prophages. The genes are ordered left to right according to their degree of enrichment in incomplete prophages; due to small numbers, gene types that constituted less than 1% of the data have been excluded. These results are summarized in the lower panels of Fig 1, which show the percent change in gene frequency between incomplete and intact prophages, that is:

$$\%\;\text{change}=\frac{100(f_{\text{inc}}-f_{\text{int}})}{f_{\text{int}}}\;\;.$$

Positive values of % change thus indicate genes that are relatively enriched in incomplete prophages, while negative values indicate genes that are preferentially lost.

To evaluate the statistical significance of these results, for each gene type we use the same number of identified genes (e.g. 317+25+14 = 356 for terminase in Data Set 1), and randomly assign the genes to one of the three prophage classes. Because intact prophages in the dataset contain more genes than incomplete prophages, we also preserve the proportion of genes assigned to each class. Thus in randomly assigning genes to prophage classes, we assign 2752/(2752+240+363) = 82% of genes to intact prophages in Data Set 1, for example, while only 363/(2752+240+363) = 11% are assigned to incomplete prophages.

We computed the percent change in gene frequency for these bootstrapped data as described above, and repeated this procedure 10,000 times. Stars in the lower panels of Fig 1 indicate % change values in the data that were lower than the 2.5 percentile or higher than the 97.5 percentile in the bootstrapped distributions.

These results reveal several features that are conserved between data sets. We note that lysis or lysin genes, as well as portal and terminase proteins, are preferentially lost in incomplete prophages. In contrast, transposase and integrase genes are substantially enriched. We explore these results further in both the computational and mathematical models described in the sections to follow.

In light of the striking enrichment of transposase genes in incomplete prophages, we examined the transposase annotations in greater detail. For each prophage in the dataset, we downloaded the coding sequences and the BLAST hits identified for each coding sequence by PHASTER [18]. We counted the number of coding sequences with a BLAST hit annotated as an insertion sequence (IS) transposase (e.g. “IS3 transposase B”), as well as those annotated as a transposase but without a BLAST hit to an IS. As a control, we also counted the total number of proteins identified as a “phage hit protein” by PHASTER in each data set.

As shown in Table 2, IS transposases account for 41.4% of the transposase sequences identified in Data Set 1, and 49.8% of those in Data Set 2. In Data Set 1, the frequency of IS transposases (calculated as the fraction of all phage proteins identified) is enriched 4.9-fold in incomplete prophages as compared to intact prophages; the frequency of non-IS transposases also increased but to a lesser degree (3.3-fold). In Data Set 2, the frequency of IS transposases increased by 10% in incomplete prophages, while non-IS transposases were reduced by 0.6%. We note that while both data sets thus displayed an enrichment of IS transposases in cryptic prophages, the degree of enrichment as far less in Data Set 2, possibly due to increased phage diversity and subsequent loss of signal in those bioinformatic data. Nonetheless, the frequency of IS transposases is enriched, in both data sets, to a greater degree than non-IS transposases. Thus, there is a strong positive association between the presence of an IS transposase, and incompleteness of the prophage. This suggests that the enrichment of transposase sequences in incomplete prophages may result from the disruption of essential prophage functions due to IS insertion; in other words, the presence of at least one transposition event has rendered the prophage cryptic.

**Table 2:** The distribution of transposase genes identified in Data Set 1 and Data Set 2.

	Number of transposase genes identified					
	Count in Data Set 1			Count in Data Set 2		
	Intact	Questionable	Incomplete	Intact	Questionable	Incomplete
IS transposase	174	34	76	459	90	109
non-IS transposase	278	37	88	464	101	99
**All phage proteins**	21054	2271	1552	19250	5097	4132

### Analytical model of prophage gene content

To investigate the preferential loss or maintenance of specific classes of phage genes in prophage sequences over time, we developed a mathematical model. The model, although simplified, allows us to find and characterize equilibrium states, and thus to understand the effects of key parameters on the long-term genetic repertoire of prophages.

The model tracks a population of bacterial genomes, which contain prophages with genes of three possible types—beneficial genes, excision genes and re-infection genes. Beneficial genes are genes that confer a selective advantage to the host, thus increasing the prophage population through vertical transmission. Biological examples of beneficial genes include host virulence factors that help the bacterial cell during colonization of its host (for example phage lambda’s *lom* gene). In contrast, excision genes are the genes involved in prophage induction into the lytic cycle, which leads to the death of the host cell. Examples of excision genes include lambda’s *O* and *P* genes, which switch on the lytic cycle. Phage induction will typically lead to bacterial cell death regardless of the quantity or quality of phage progeny. Phage progeny success is determined by the phage’s re-infection genes, comprising the genes required for phage genome replication, packaging, lysis, transmission to a new host, and reestablishment of lysogeny. This re-infection class, in particular, includes a large number of genes of different function; their net effect, taken together, is to increase the prophage population through horizontal transmission.

In the simplified model, we consider a full prophage as one containing just three ‘genes’, one of each class. Here, we can think of a ‘gene’ as a full functional complement of the underlying sequences required for each function. We denote the frequency of bacterial genomes that contain full prophages at time *t* as *P*_111_(*t*). More generally, we use the notation *P*_*ber*_ to represent the frequency of bacterial genomes containing prophages with (1) or without (0) the beneficial, excision or re-infection genes respectively. For completeness, the model must also include a population *P*_000_ corresponding to bacterial genomes in which the prophage has been completely lost. Note that in the individual-based simulation approach in the next section, we will both expand these gene classes and include multiple genes per class.

The analytical model includes the following processes:

**Degradation**: Each gene in each prophage in the population is lost (gene deletion) at rate *r*_*D*_. For example, the frequency of *P*_111_ is lost at overall rate 3*r*_*D*_, contributing at rate *r*_*D*_ to each of the populations *P*_011_, *P*_101_ and *P*_110_. We note that *r*_*D*_, like any of the rates in this model, may be expressed in any time unit; in previous work we have used the rate per prophage “lifetime”, that is, the mean time between lysogeny and induction [17]. However in this contribution we will use the analytical model to examine equilibria and stability conditions, and thus the time scale is arbitrary (only the relative magnitudes of the rates will come into play).

**Induction**: If a bacterial genome contains a prophage which carries the excision gene, the prophage induces at rate *r*_*I*_ and the bacterium is lost from the population.

**Re-infection**: Prophages that carry both the excision and re-infection genes (*P*_111_ and *P*_011_) reproduce (create copies of themselves in new bacterial genomes), through lysis, re-infection and lysogeny, at rate *r*_*L*_.

**Selection**: To model the potential selective benefit conferred by the prophage, we assume that bacterial populations that carry the beneficial prophage gene grow at per capita rate *r*_*S*_.

**Fig 2:**
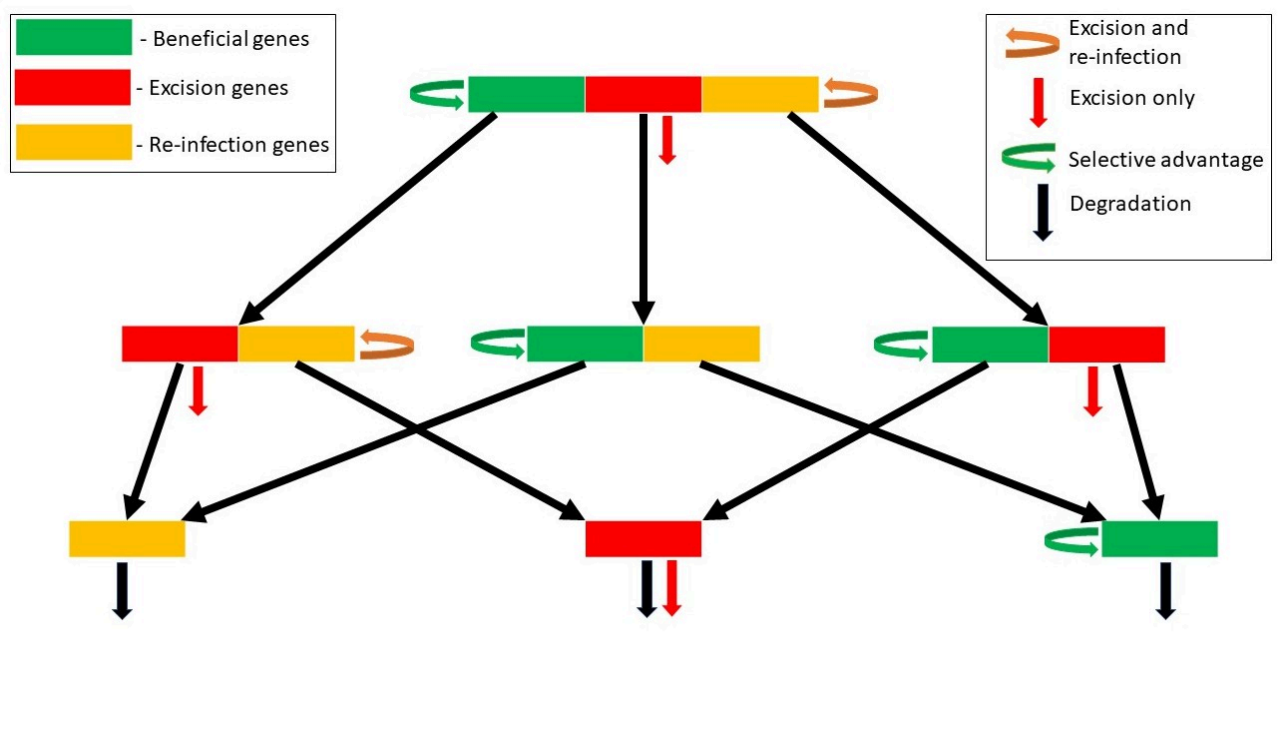
Schematic diagram of the mathematical model.

These assumptions yield the following system of ordinary differential equations, illustrated as a schematic in Fig 2:

$$\begin{array}{ccc} \frac{dP_{1\;1\;1}}{dt} & = & \left(r_{L}+r_{S}-3r_{D}-r_{I}\right)P_{1\;1\;1}-\phi P_{1\;1\;1}\\ \frac{dP_{0\;1\;1}}{dt} & = & \left(r_{L}-2r_{D}-r_{I}\right)P_{0\;1\;1}+r_{D}P_{1\;1\;1}-\phi P_{0\;1\;1}\\ \frac{dP_{1\;0\;1}}{dt} & = & \left(r_{S}-2r_{D}\right)P_{1\;0\;1}+r_{D}P_{1\;1\;1}-\phi P_{1\;0\;1}\\ \frac{dP_{1\;1\;0}}{dt} & = & \left(r_{S}-2r_{D}-r_{I}\right)P_{1\;1\;0}+r_{D}P_{1\;1\;1}-\phi P_{1\;1\;0}\\ \frac{dP_{0\;0\;1}}{dt} & = & \left(-r_{D}\right)P_{0\;0\;1}+r_{D}P_{0\;1\;1}+r_{D}P_{1\;0\;1}-\phi P_{0\;0\;1}\\ \frac{dP_{0\;1\;0}}{dt} & = & \left(-r_{D}-r_{I}\right)P_{0\;1\;0}+r_{D}P_{0\;1\;1}+r_{D}P_{1\;1\;0}-\phi P_{0\;1\;0}\\ \frac{dP_{1\;0\;0}}{dt} & = & \left(r_{S}-r_{D}\right)P_{1\;0\;0}+r_{D}P_{1\;0\;1}+r_{D}P_{1\;1\;0}-\phi P_{1\;0\;0}\\ \frac{dP_{0\;0\;0}}{dt} & = & r_{D}\left(P_{1\;0\;1}+P_{0\;1\;0}+P_{0\;0\;1}\right)-\phi P_{0\;0\;0}\;\;. \end{array}$$ (1)

Here, terms involving *ϕ* simply ensure that the frequencies sum to unity at all times, with *ϕ* defined as:

$$\phi =\left(r_{L}+r_{S}-r_{I}\right)P_{1\;1\;1}+\left(r_{L}-r_{I}\right)P_{0\;1\;1}+r_{S}P_{1\;0\;1}+\left(r_{S}-r_{I}\right)P_{1\;1\;0}-r_{I}P_{0\;1\;0}+r_{S}P_{1\;0\;0}\;\;.$$

A detailed analysis of the equilibria and stability of this 8-dimensional model is provided in the S2 Appendix. This rigorous analysis allows us to demonstrate that only four long-term outcomes are possible in this system: (1) equilibrium *E*_0_, in which all prophage genes are lost; (2) equilibrium *E*_*B*_, in which both excision and re-infection genes are lost, but beneficial genes persist. This equilibrium reflects complete domestication of the prophage; (3) equilibrium *E*_*ER*_, in which beneficial genes are lost but both excision and re-infection genes persist. This corresponds to a completely parasitic prophage that does not contribute to host fitness; (4) equilibrium *E*_*A*_, in which all three types of genes persist. Note that these four equilibria are a subset of all possible outcomes; it is not possible, for example, for prophages that contain only the excision and beneficial genes to persist in the long term.

As described in the S2 Appendix, the mathematical model also allows us to identify two critical conditions that are sufficient to determine the long-term behaviour of the prophage gene distribution.

Condition 1: *r*_*S*_ > *r*_*D*_. We can think of *r*_*S*_ as the rate at which a beneficial gene produces a new copy of itself, while *r*_*D*_ is the rate at which a beneficial gene is lost, through mutational degradation. Thus *r*_*S*_ > *r*_*D*_ implies that on average, a beneficial gene makes more than one copy of itself before it is lost: beneficial genes persist.

Condition 2: *r*_*L*_ > 2*r*_*D*_ + *r*_*I*_. Similarly, the combination of an excision and a re-infection gene, co-occurring on a prophage, is able to produce a new copy of itself at rate *r*_*L*_. These genes may be lost through induction, but also lost if either gene is degraded by mutation, so the total rate of loss is 2*r*_*D*_ + *r*_*I*_. Thus this gene combination can persist if *r*_*L*_ > 2*r*_*D*_ + *r*_*I*_.

The predicted behaviour of the mathematical model can therefore be summarized as shown in Table 3.

**Table 3:** Conditions determining which classes of prophage genes persist long-term.

**Long-term prediction** Condition	B genes do not persist *r*_*S*_ < *r*_*D*_	B genes persist *r*_*S*_ > *r*_*D*_
ER genes do not persist *r*_*L*_ < 2*r*_*D*_ + *r*_*I*_	**extinction** *E*_0_	**domestication** *E*_*B*_
ER genes persist *r*_*L*_ > 2*r*_*D*_ + *r*_*I*_	**parasitism** *E*_*ER*_	**persistence** *E*_*A*_

**Fig 3:**
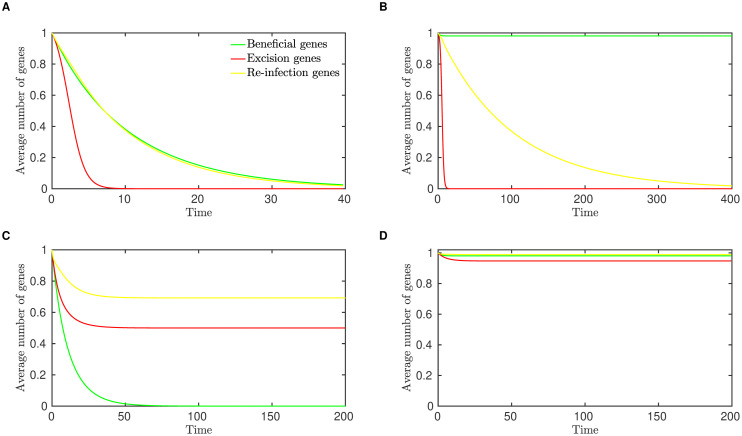
**Numerical integration of the analytical model, showing the system of equations (Eq 1) converging toward four possible equilibria:** (A) **Extinction**, *E*_0_(*r*_*S*_ = 0.01, *r*_*D*_ = 0.1, *r*_*L*_ = 0.2). (B) **Domestication**, *E*_*B*_ (*r*_*S*_ = 0.52, *r*_*D*_ = 0.01, *r*_*L*_ = 0.2). (C) **Parasitism**, *E*_*ER*_ (*r*_*S*_ = 0.02, *r*_*D*_ = 0.1, *r*_*L*_ = 1.3). (D) **Persistence**, *E*_*A*_ (*r*_*S*_ = 0.52, *r*_*D*_ = 0.01, *r*_*L*_ = 1.2). In all cases, *r*_*I*_ = 1.

In Fig 3, we illustrate the approach of the system of ordinary differential equations (Eq 1) to each of these four equilibrium states, for appropriate parameter values. To simplify the presentation, we plot the the average number of genes of each type carried per bacterial genome, where for example the average number of beneficial genes per genome is given by *P*_111_ + *P*_110_ + *P*_101_ + *P*_100_. Equivalently, this is the fraction of genomes that carry the beneficial prophage gene.

The numerical values of the parameters for the domestication equilibrium, Fig 3B, have been taken directly from our previous work, in which detailed model selection and model fitting were performed to determine which evolutionary processes best explain the prophage length distribution [17]. We refer the interested reader to that contribution for a more nuanced discussion of these parameter values, including comparisons with the literature.

### Gene repertoire simulations

As a third approach to understanding the gene content of prophage sequences, we developed a computational simulation in which individual prophage sequences are represented as bit strings, and an entire population of bacterial genomes is simulated over time. Thus, rather than use differential equations to describe the expected average behaviour of the population, we use an individual-based simulation in which each prophage is subject to stochastic events such as the mutational loss of a specific gene, reproduction of the host cell, or induction. Unlike the analytical model, this computational approach allows us to examine prophages with various numbers of genes contributing to different functions, and in particular to examine the distribution of prophages of varying length. The code for these simulations was developed in C++ and is available at: https://github.com/MathBioInfo/Computational-Model.

Here, we assume that prophages exist in a population of bacterial genomes, where the bacterial population size is subject to population regulation through carrying capacity *K*. However, each bacterial genome may or may not contain a prophage, and thus the prophage population size can vary. We further assume that prophages confer immunity to re-infection by the same phage, and so each bacterial genome carries at most one prophage sequence. To simplify the analysis, we assume that immunity is conferred by cryptic (degraded) prophages as well as full prophages. Each prophage sequence may contain genes of the following four types: excision genes, re-infection genes, beneficial genes and neutral genes. A full prophage carries *n*_*E*_ excision genes, *n*_*R*_ re-infection genes, *n*_*B*_ beneficial and *n*_*N*_ neutral genes. Thus for example if *n*_*E*_ + *n*_*R*_ + *n*_*B*_ = 10, a full prophage is represented as ‘1111111111’. We track the presence or absence of each gene in each prophage sequence; a degraded prophage, for example, might be represented as ‘1001111001’.

A discrete timestep in the simulation corresponds to a bacterial generation time. The rates of the underlying processes, however, are expressed in units of the “prophage generation time”, that is, the average time that a single prophage sequence is maintained in a bacterial genome before induction [17]. Since the bacterial generation time, Δ*t*, is much shorter than the prophage generation time, if a process occurs at rate *r* per prophage generation, the probability that it occurs in timestep Δ*t* is small and well-approximated by *r*Δ*t*.

We use a Wright-Fisher model of the bacterial population dynamics in discrete time, modified slightly to allow for stochastic fluctuations in the bacterial population size. Thus in each discrete timestep, a pool of new bacterial genomes is created as described below. This pool is then subject to random sampling to determine the next generation (see “Population regulation”).

The following processes are included in the model, with parameters as described in Table 4:

**Table 4:** Parameters of the computational model.

Parameter	Description
*n*_*B*_	number of beneficial genes
*n*_*E*_	number of genes necessary for excision
*n*_*R*_	number of genes necessary for re-infection
*n*_*N*_	number of neutral genes
*r*_*D*_	rate of loss through mutational degradation
*r*_*I*_	rate of loss through induction, excision and host death
*r*_*L*_	rate of increase through lysis, reinfection and lysogeny
*r*_*T*_	rate of loss through TE disruption
*r*_*S*_	selective advantage to host cell if prophage carries all beneficial genes

**Degradation**: In each time step, before bacterial reproduction, each gene in each prophage in the population is removed (gene deletion, ‘1’ changed to ‘0’) with probability *r*_*D*_Δ*t*.

**Induction**: If a prophage carries all *n*_*E*_ excision genes, it may induce with probability *r*_*I*_Δ*t*. When a prophage induces, the bacterial genome carrying that prophage is removed from the population before bacterial reproduction. We thus assume that all *n*_*E*_ excision genes are required for excision and death of the host cell.

**Selection**: To model bacterial reproduction, each bacterial genome is copied to the pool from which the next generation will be drawn. An additional copy of each genome is added to the pool with probability *r*_*S*_Δ*tn*_*b*_/*n*_*B*_. Here *n*_*b*_ is the number of beneficial genes carried by prophage in the genome, and *r*_*S*_ is the maximum selective benefit provided to the host cell if the prophage contains all *n*_*B*_ beneficial genes.

**Re-Infection**: To simulate the process of lysis followed by re-infection and lysogeny, a copy of any genomes that carry prophages with *n*_*E*_ excision genes *and* all *n*_*R*_ re-infection genes may also be added to the pool of new bacterial genomes with probability *r*_*L*_Δ*t*. Thus, full complements of both the excision and re-infection genes are required to reinfect.

**Population regulation**: To regulate the population size, if the population size in the bacterial pool *N*, is greater than the bacterial carrying capacity, *K*, each bacterial genome is copied into the subsequent generation with probability *K*/*N*.

While all of our simulation studies include the processes described above, in some simulations we also explored the impact of disruption by transposable elements (TEs, such as bacterial insertion sequences) as follows.

**TE disruption**: Motivated by the observed frequencies of IS transposase sequences in incomplete prophages, we include the possibility of TE disruptions in prophage genes. For each gene in each timestep, a TE disruption may occur with probability *r*_*T*_Δ*t*. When this occurs, we assume that gene function has been disrupted: if a beneficial gene has been disrupted, the gene confers no benefit to the host thereafter; if a gene required for excision or re-infection is disrupted, the prophage is no longer able to kill the host or re-infect respectively. Thus TE disruptions have the same effect as gene deletions, but leave a signature of TE sequences in the prophage genome.

We wondered whether it was reasonable to include TE disruptions in the model, since their rates might be negligible relative to mutational degradation. Rates of base pair substitutions in *E. coli* K12 have been estimated to be on the order of 2 × 10^−10^ per nucleotide, per generation [19]. Multiplying by 1.2 kbp per prophage gene [17] yields an estimate of 2.4 × 10^−7^ base pair substitutions, per prophage gene, per bacterial generation. Presumably only a fraction of base pair substitutions result in loss of function. In addition, small indels are estimated to occur at about one tenth of this rate [19]. Thus taking in *E. coli* as a model organism, rates of prophage gene degradation through mutation (base pair substitution and short indels) might occur on the order of 10^−7^ or 10^−8^ per gene per generation.

In comparison, rates of transposition, for 5 insertion sequences in *E. coli* K12, have been estimated to be about 1 × 10^−5^ per element per generation [20]; this includes both copy-and-paste and cut-and-paste transpositions. The ancestral genome in this mutation accumulation study carried a total of 33 copies of these ISs, yielding an overall transposition rate of 3.3 × 10^−4^ transpositions per generation. Given that a typical prophage gene comprises 1.2 kbp [17] of a 4.6 Mbp *E. coli* genome, we arrive at an estimated transposition rate of 8.6 × 10^−8^ per prophage gene, per bacterial generation, similar to our estimate for gene loss through mutational degradation.

#### Gene content of active temperate phage

We used phage lambda’s genome architecture as a model for the number of excision, beneficial, and reinfection genes in a temperate phage genome (see Fig 1 in [21]). Lambda has long been a model system for the study of lytic-lysogeny cycles, phage genome arrangement, and phage evolution [22].

Excision genes: Lambda’s excision genes are those that switch phage gene expression to the lytic cycle. Corresponding to the early right operon (6.5 kbp), these excision genes make up approximately 13.3 percent of the phage genome.

Beneficial genes: Lambda carries several genes thought to confer benefit to the bacterial host during lysogeny. These include *cI*, *rexA*, *rexB*, *sieB*, *lom*, and *bor*, comprising ∼3.7 kbp total, about 7.6 percent of the genome.

Reinfection genes: The rest of the lambda genome contains genes that allow a phage to form viable progeny capable of reinfecting other cells: phage particle production, packaging, lysis, and lysogeny. These genes include about 38.4 kbp, ∼79 percent of the genome. Most of these genes are contained in the late Operon (∼27 kbp, phage particle production) and the early left operon (∼13 kbp, lysogeny). The host-beneficial genes encoded in those operons (*sieB*, *lom*, *bor*, ∼1.6 kbp total) are included instead in the beneficial genes category discussed above.

We note that not all lambda genes have been fully characterized. For example, *lom* and *bor* are thought to be host-beneficial during lysogeny, but more work is needed to establish the host fitness components. We also note that not all excision and reinfection phage genes are likely essential.

Taken together, these gene frequencies motivated the choice to model a full prophage genome in the ratio 1:1:8 for beneficial:excision:re-infection genes. In addition to these genes, in some simulations we also included neutral genes as a control.

## Results

**Fig 4:**
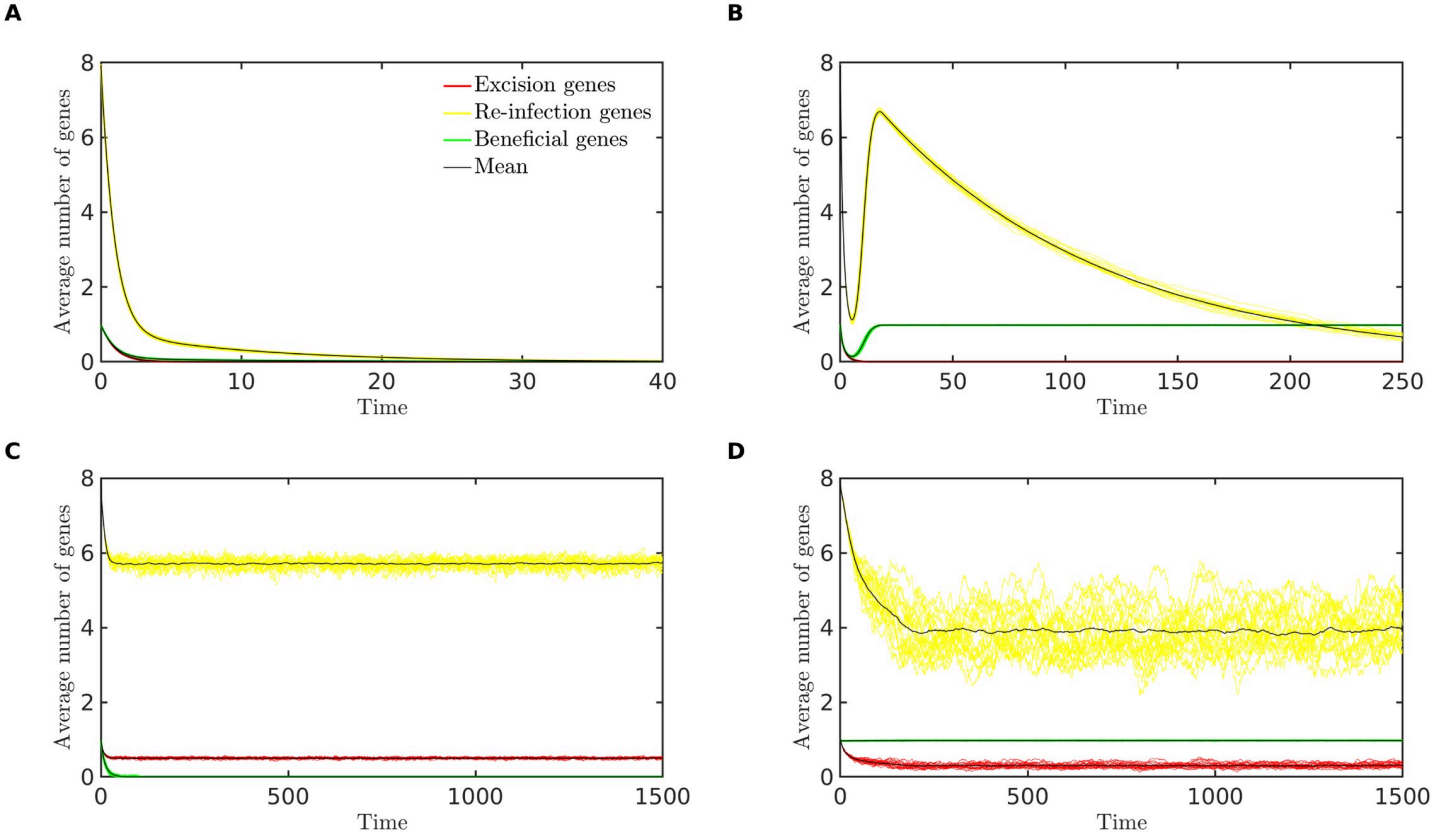
**Simulations results showing the approach to four possible long-term outcomes:** (A) **Extinction** (*r*_*S*_ = 0.01, *r*_*D*_ = 0.1, *r*_*L*_ = 0.2). (B) **Domestication** (*r*_*S*_ = 0.52, *r*_*D*_ = 0.01, *r*_*L*_ = 0.2). (C) **Parasitism** (*r*_*S*_ = 0.02, *r*_*D*_ = 0.1, *r*_*L*_ = 2.0). (D) **Persistence** (*r*_*S*_ = 1.5, *r*_*D*_ = 0.05, *r*_*L*_ = 1.5). In all cases, *r*_*I*_ = 1, *r*_*T*_ = 0, *n*_*B*_ = 1, *n*_*E*_ = 1 and *n*_*R*_ = 8. The average number of genes of each type, per prophage, is plotted against time, for 20 randomly chosen replicate simulations (colored lines) and for the mean across 100 replicate simulations (black lines). For each replicate, a population of 10,000 bacterial genomes was simulated for 20,000 bacterial generations, over which time each of these long-term outcomes remained stable; here we illustrate the initial approach to these long-term states.

The computational model shows excellent agreement with the results pertaining to the bioinformatic analysis and analytical model shown in the previous sections. Fig 4 illustrates that as predicted by the analytical model, the long-term behaviour of the simulation displays four possible outcomes for the prophage: extinction, domestication, parasitism (loss of genes that benefit the host but retention of genes necessary for infection) or persistence (of all gene types). Although omitted for brevity, it is straightforward to derive conditions similar to those provided in Table 3 which predict the loss or retention of each gene type. For example with 1 excision gene and 8 re-infection genes, the ‘ER’ (excision-reinfection) function can be lost by a mutation in any of these 9 genes, so the overall rate of loss is 9*r*_*D*_ + *r*_*I*_, while the rate of gain is *r*_*L*_.

We note, however, that the long-term outcomes obtained in the simulations are more complex than the equilibria of the analytical model. In Fig 4D, for example, the average number of re-infection genes has been reduced to less than four genes, while eight genes (a full complement) are required for functionality. If a single prophage sequence contains only four re-infection genes, these genes are effectively neutral and should be further degraded over time. This apparent contradiction is explained by considering the distribution of prophage genes among prophages of different lengths, as described below.

**Fig 5:**
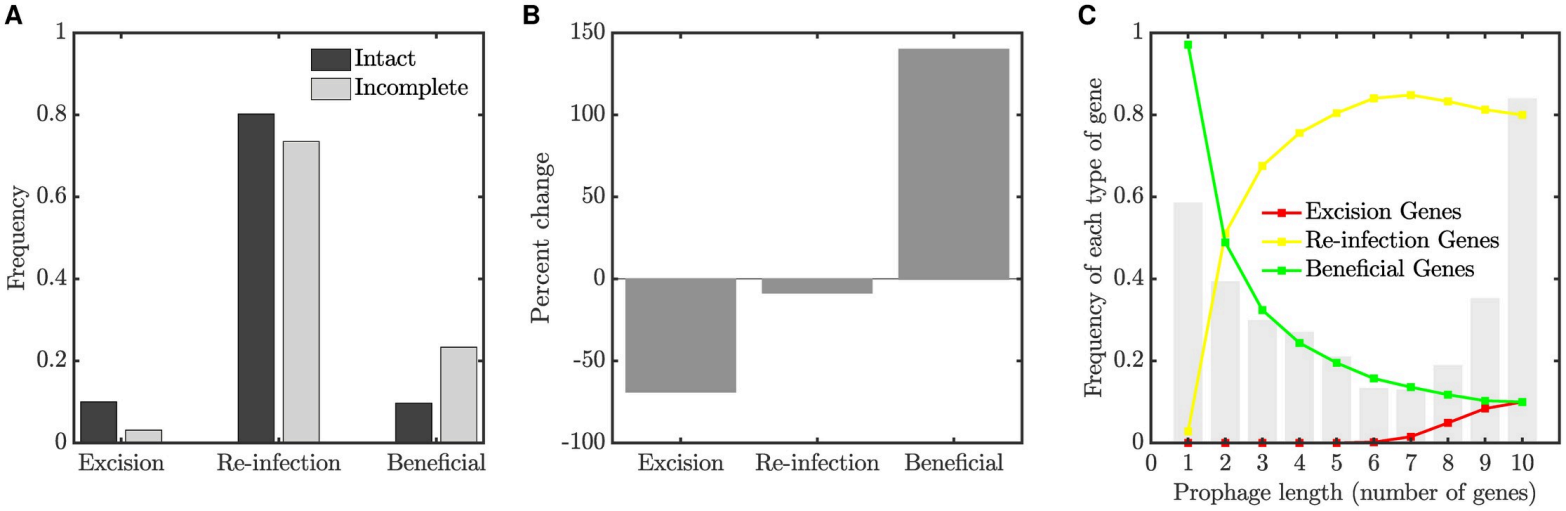
Gene frequencies in intact and incomplete prophages. (A) Frequency of genes of each type in intact and incomplete prophages, for the computational model simulated at the persistence equilibrium (see text for details). (B) Percent change in gene frequency from intact to incomplete. (C) A histogram of prophage lengths (grey bars), as well as the frequency of gene classes at each length. We find a bimodal distribution of prophage sizes, with smaller prophages losing the excision and re-infection genes but retaining the beneficial gene. A population of 10,000 prophages was simulated for 20,000 generations.

We investigated how gene content and genome length vary within a prophage population in the long term. We were interested in whether our simulations resulted in the evolution of a bimodal genome distribution as found bioinformatically, and if the same type of genes were enriched in incomplete prophages. To do this, we simulated the prophage population with parameter values as described in panel D of Fig 4 for 20,000 generations, and then compared the gene content of prophages of different lengths. We define all prophages as either “intact” or “incomplete”: an intact prophage carries all the genes necessary for excision and re-infection, whereas if any of these genes is missing, the prophage is incomplete. Fig 5A shows the frequency of each type of gene in intact and incomplete prophages; the percent change in incomplete prophages, as compared to the baseline of an intact prophage, is shown in panel B. We find that genes involved in excision and re-infection are preferentially lost in incomplete prophages.

We also observe a bimodal distribution of prophage lengths in these simulations (Fig 5C, grey bars). The gene frequencies at each length (Fig 5C, solid lines) reveal that the smallest prophages have become domesticated, that is, they retain only the gene that benefits the host.

Finally, we note that a large fraction of full prophages is maintained in the long term (right-most bar in Fig 5C). These prophages are continually replenished by both re-infection and host cell replication. Once a prophage has lost a single re-infection gene, however, the remaining genes in this class are effectively neutral and the prophage would become progressively shorter due to further gene loss. The left-most bar reveals that a large fraction of fully domesticated prophages is also maintained due to host cell replication. Thus, while the average number of re-infection genes in the population is around four (see Fig 4D), most prophages carry either all the re-infection genes, or none of them.

**Fig 6:**
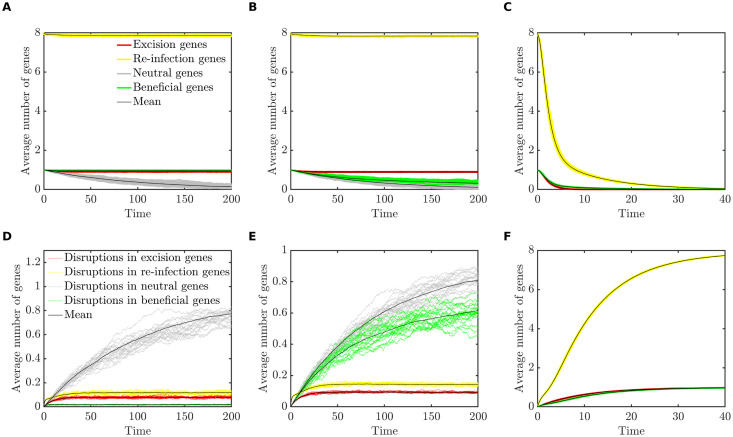
The effect of TE disruptions on the long-term outcome for prophage sequences. In panels A through C, the average number of genes of each type per prophage is plotted against time. As the transposition rate is increased relative to the selective advantage of the beneficial gene, the long-term prediction for the population changes from persistence (panel A) to parasitism (panel B) and finally to extinction (panel C). Panels D through F show the average number of TE disruptions sustained in genes of each type versus time. TEs accumulate in neutral genes but are limited in functional genes due to purifying selection. Results are plotted for 20 randomly chosen replicate simulations (colored lines) and for the mean across 100 replicate simulations (black lines). For each replicate, a population of 10,000 bacterial genomes was simulated for 20,000 bacterial generations, over which time each of these long-term outcomes remained stable; here we illustrate the initial approach to these long-term states. Parameter values are: (A and D) *r*_*S*_ = 0.52, *r*_*T*_ = 0.009. (B and E) *r*_*S*_ = 0.01, *r*_*T*_ = 0.01. (C and F) *r*_*S*_ = 0.002, *r*_*T*_ = 0.1. In all cases, *r*_*I*_ = 1, *r*_*L*_ = 1.2, *r*_*D*_ = 0.001, *n*_*B*_ = 1, *n*_*E*_ = 1, *n*_*R*_ = 8 and *n*_*N*_ = 1.

Fig 6 illustrates the effect of adding transposable element disruptions to the computational model. In panel A, despite TE disruptions, the prophage population persists and retains all genes. Here we have also added a single neutral gene, which has no effect on fitness, for comparison (grey line). Panel D shows the average number of TE disruptions sustained in each type of gene; TEs accumulate in neutral genes but their presence in functional genes is minimized by purifying selection. Panels B through F show similar results, except that the rate of TE disruption, *r*_*T*_, and the selective advantage, *r*_*S*_, are altered. Increasing the transposition rate has the same qualitative effect as increasing the mutation rate, *r*_*D*_, in Table 3; the long-term outcome can change from persistence (panel A) to either parasitism (panel B) or domestication, depending on the value of *r*_*S*_, and then ultimately to extinction (panel C) as *r*_*T*_ increases.

Again, we simulated the prophage population with parameter values as described in panel D of Fig 4 for 20,000 generations, but including TE disruptions (*r*_*T*_ = 0.002), comparing the gene content of intact and incomplete prophages. Using the strict definition of “intact” described above (an intact prophage carries all the genes necessary for excision and re-infection, whereas if any of these genes is missing, the prophage is incomplete), transposase genes were enriched nearly 105-fold in incomplete prophages (see S3 Appendix).

This result may be artificially inflated by the fact that only the single beneficial gene can sustain a TE disruption in an intact prophage in our simulations. In reality, algorithms such as PHASTER are not able to classify prophages as intact based on the certainty that they contain a full complement of functional phage genes. Instead, approximate metrics are used, based for example on the number of identified phage genes in close proximity in the sequence [18]. For a better comparison with the data shown in Fig 1, we therefore examined results if prophages that contained 80% or more of the possible prophage genes were classified as “intact”; prophages with less than 80% were classified as incomplete.

**Fig 7:**
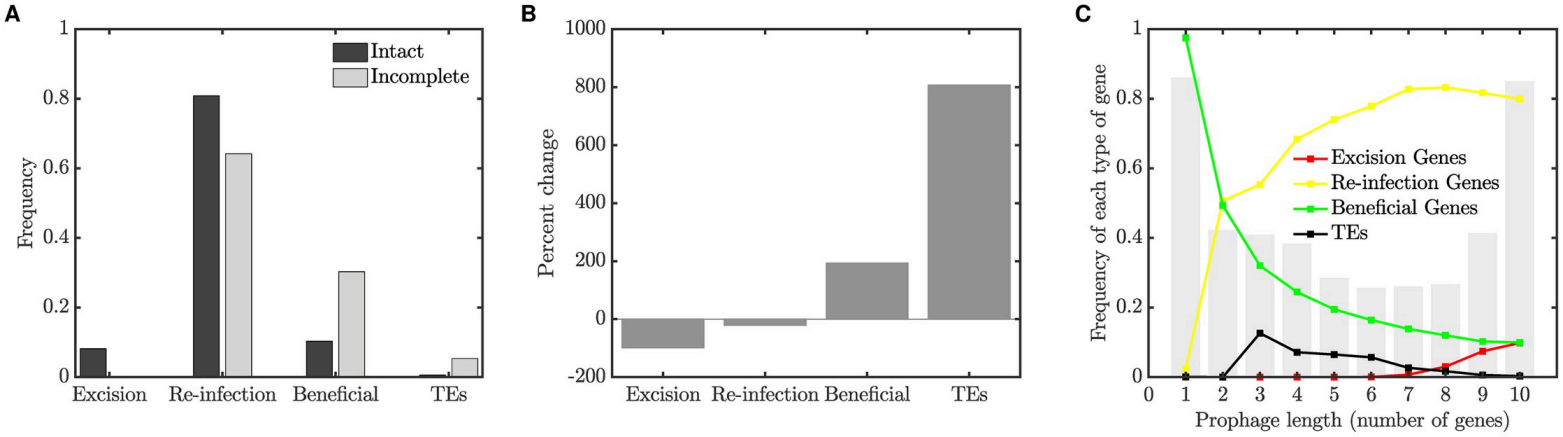
Gene frequencies in intact and incomplete prophages, when TEs are included. (A) Frequency of genes of each type in intact and incomplete prophages, for the computational model simulated at the persistence equilibrium with TE disruptions (see text for details). (B) Percent change in gene frequency from intact to incomplete. (C) A histogram of prophage lengths (grey bars), as well as the frequency of gene classes at each length. We find a bimodal distribution of prophage sizes, with TEs accumulating in prophages of intermediate lengths. In all cases *r*_*S*_ = 1.5, *r*_*L*_ = 1.5, *r*_*D*_ = 0.05 and *r*_*T*_ = 0.002. A population of 10,000 prophages was simulated for 20,000 generations.

Fig 7A shows the frequency of each type of gene in intact and incomplete prophages classified in this way; the percent change in incomplete prophages, as compared to the baseline of an intact prophage, is shown in panel B. Again we see that genes involved in excision and re-infection are preferentially lost, beneficial genes are preferentially maintained, and transposase genes are substantially enriched in shorter prophages. These conclusions were not sensitive to the fraction of genes used as the threshold to identify intact prophages (see S3 Appendix).

Fig 7C shows a histogram of prophage lengths (grey bars), along with the gene frequency for each gene type, for prophages of each length. A bimodal distribution of prophage lengths is again demonstrated, with the smallest prophages becoming domesticated, that is, retaining only the gene that benefits the host. We note that transposase genes accumulate in prophages of intermediate length, but are absent from the smallest prophages.

### Confirmation of predicted transposase distribution

**Fig 8:**
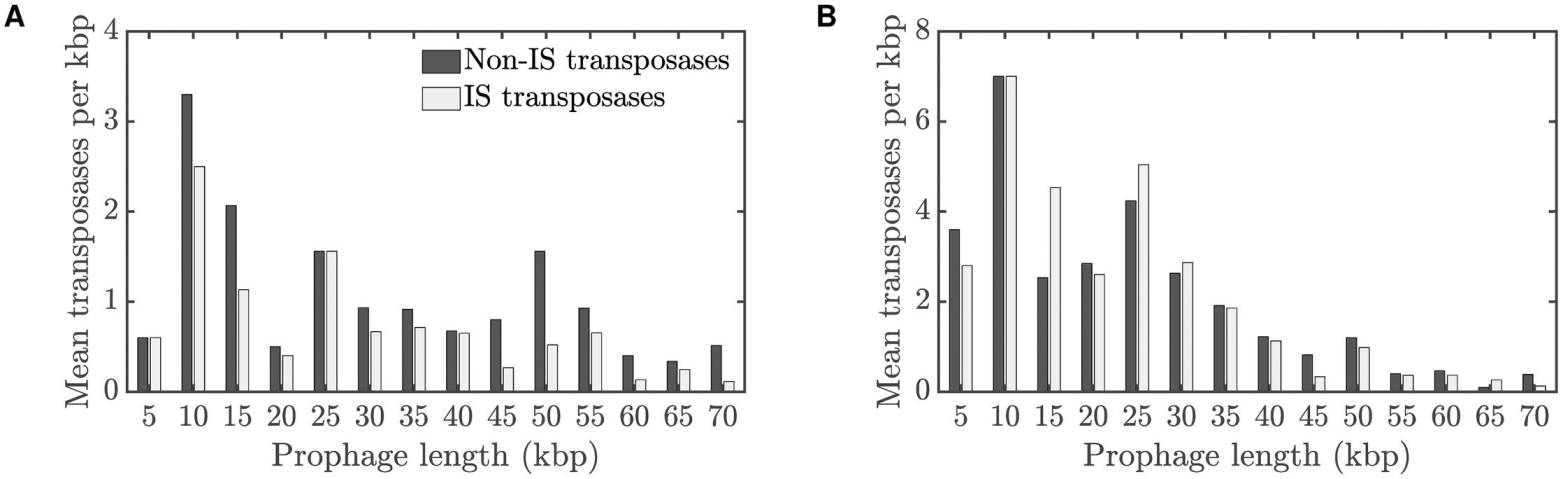
**Average number of transposases per kbp of prophage sequence, for prophages of different lengths:** (A) Average number of transposases per kbp of prophage sequence for Data Set 1. (B) Average number of transposases per kbp of prophage sequence for Data Set 2.

Motivated by this computational prediction of the distribution of transposase genes (black line in Fig 7C), we returned to the bioinformatics data to test whether the prediction was borne out, recording the number of transposases per prophage and stratifying these counts by prophage length. In Fig 8, we show the distribution of both non-IS transposase and IS transposase sequences versus prophage length. Here, the number of prophages has been normalized by the length of the prophage sequence, so the *y*-axis represents the number of transposases per kbp. These results show a clear enrichment of transposase sequences in intermediate-length prophages; in addition, the degree of enrichment shows an increasing trend as prophage length decreases, for all but the shortest prophage lengths. These qualitative features show striking agreement to the distribution of TEs predicted in Fig 7C.

For reference, we also provide distribution of the number of transposases per prophage in the Supplementary Material (S4 Appendix).

## Discussion

We bring three lines of evidence to bear on the diverse genetic repertoire of active and cryptic prophages. First, we examine the distribution of over 7000 gene annotations from sequenced prophages to demonstrate that genes involved in lytic function—structural genes such as plate, capsid and portal genes, as well as lysis, lysin and terminase genes—are preferentially lost in incomplete (presumably cryptic) prophages. In constrast, three gene classes are enriched: tail fiber, integrase and transposase genes (Fig 1C and 1D).

Secondly, a simplified mathematical model predicts that depending on the balance among dynamic processes such as the rates of lysis and infection, selection and mutational degradation, only four long-term outcomes are sustainable for prophage sequences: the maintenance of an active prophage that also carries host-beneficial genes, the maintenance of an active but completely parasitic prophage, domestication, or extinction (Fig 3 and Table 3). Together, these outcomes represent the evolution of prophage sequences along a parasitism-mutualism continuum.

Thirdly, a computational approach (individual-based simulation) examines the genetic repertoires of evolved prophages with genomes of different lengths. The computational model predicts a bimodal distribution of prophage lengths, as observed in a number of recent bioinformatic studies [13–16], and consistent with our recent analytical predictions regarding the interplay of selection and mutation on the prophage length distribution [17].

The computational model also demonstrates that genes involved in excision and re-infection are preferentially lost in shorter prophages (Figs 5 and 7), consistent with the loss of lytic-cycle specific genes (such as structural, lysis and terminase genes) observed our bioinformatic analyis (Fig 1). This result is intuitively appealing since selection at the level of the host is in most contexts expected to favor evolution away from phage parasitism through the loss of intact lytic-cycle alleles. The preferential loss of terminases we observed may also be related to gene length; terminase genes tend to be larger than most prophage genes (approximately 2kbp compared to the more typical phage gene length of 1kbp), potentially rendering them more susceptible to deletions.

As well as revealing these gene losses, our bioinformatic analysis also supported a previous finding [13] that some genes are significantly enriched in shorter prophages. In our data, these enriched genes included transposases, phage tail genes, and integrases. Consistent with these data, transposases preferentially accumulated in cryptic prophages in our simulation studies (Fig 7), where transposable elements disrupted gene function and left an identifiable transposase gene sequence as a signature. Motivated by the enrichment of transposable elements in intermediate-length prophages observed in the simulation, we were able to return to the bioinformatics data and confirm this prediction (Fig 7C, black line, as confirmed in Fig 8).

A second group of enriched genes, tail fibre genes, would be classified as re-infection genes important to the parasitic lytic cycle, so they were not predicted to be enriched in cryptic prophages in our simulations. Bobay et al. [13] hypothesize that tail genes may be domesticated by bacterial hosts through the longer-term processes of co-option and the evolution of novel functions, for example the evolution of bacterial tailocin toxins from phage tail ancestors. For domestication of tail genes in this way, such processes would presumably require a specific combination of multiple accumulated mutations and the appropriate selective environment for a novel function to emerge [23]. These conditions were well beyond the scope of our models here, but including the additional complexity of domestication via the accumulation of *de novo* adaptive mutations is an interesting idea for future work.

The enrichment of integrase genes in short prophages was an unexpected result of our bioinformatic analysis. Prophages typically possess integrase genes that facilitate integration into the host genome [24–27], making the host vulnerable to temperate phage infection and, later, lytic phage reproduction. Therefore, we expected selection to act against functional integrase genes.

We suggest that integrase genes may be maintained through the evolution of phage-like genetic selfish elements, such as satellite phages and molecular parasites that don’t require the full complement of phage lytic cycle genes but benefit from horizontal transfer among hosts. When foreign DNA enters a host cell, integrase can mediate the process of recombination, thereby facilitating horizontal gene transfer. The transfer of integrase genes along with their neighboring prophage genes would then result in significant enrichment of integrase genes in incomplete prophages. Our computational model did not include horizontal transmission of partial prophages, but investigating this hypothesis is a clear avenue for future work. An alternative, but not mutually exclusive, biological explanation for the enrichment of integrase genes is that these genes tend to be located at the border of prophages, which could help their retention through deletion shielding: if large deletions removed integrase genes, those deletions would be selected against if they also deleted bacterial genes in the adjacent bacterial chromosome [13]. Finally, we note that although the presence of an integrase gene is often an inclusion criterion for prophage detection, the PHASTER algorithm considers the presence of a wide range of phage genes, including integrases, and thus detection bias is unlikely to explain these results.

A clear limitation of our approach is that in order to develop tractable models of prophage gene content, we have neglected the complex population dynamics of uninfected hosts, lysogens, and free phage. In reality, multiple factors such as the availability of sensitive hosts, super-infection exclusion, and the lysis-lysogeny decision will play important roles in these dynamics [28–31], and will also affect selective pressures on the phage and thus gene content. We also note that while our approach includes benefits conferred to the host through individual prophage genes, we neglect several demonstrated benefits that only intact prophage may confer to their hosts, including the use of prophages as ecological weapons [32], agents of horizontal gene transfer [33], or engineers of biofilm structure and function [34].

Overall, we predict that the fundamental evolutionary forces of mutation, positive and negative selection maintain prophages of differing lengths and diverse gene content along the parasitism-mutualism continuum, and offer explanations for both the enrichment and loss of specific gene functions in cryptic prophages. A deeper understanding of these complex and multi-level evolutionary dynamics may have broad appeal to those interested in the critical roles of prophages in the spread of antibiotic resistance genes and virulence determinants.

## Supporting information

S1 Appendix Supplemental methods. (PDF)

S2 Appendix Fixed point and stability analysis of system of ordinary differential equations (Eq 1). (PDF)

S3 Appendix Transposase enrichment in incomplete prophages. (PDF)

S4 Appendix Transposase distribution. (PDF)
