# Supplementary material for: Evolution along the parasitism-mutualism continuum determines the genetic repertoire of prophages
Source: PLoS Comput Biol. 2020 Dec 4;16(12):e1008482. doi: 10.1371/journal.pcbi.1008482 (PMC7744054; doi:10.1371/journal.pcbi.1008482)
Supplement: S1 Appendix — (PDF) [file pcbi.1008482.s001.pdf]

Evolution along the parasitism-mutualism continuum determines the genetic repertoire of prophages

Amjad Khan[1], Alita R. Burmeister[2, 3], Lindi M. Wahl[1,*]

1 Department of Applied Mathematics, Western University, London, Ontario, Canada.
2 Department of Ecology and Evolution, Yale University, New Haven, Connecticut, USA.
3 BEACON Center for the Study of Evolution in Action, East Lansing, Michigan, USA.

* lwahl@uwo.ca

S1 Appendix. Supplemental Methods

Data

We investigated the annotations of 53,356 genes from two previously published and publicly available data sets, referred to as Data Set 1 [1] and Data Set 2 [2]; see Table 1 for details.

Table 1. Summary of Data Set 1 and Data Set 2.

Data Set	# of genomes	# of prophages	# of genes	Bacterial species	Version	Reference
1	85	624	24,877	*E. Coli* (474 prophages) and *S. Enterica* (150 prophages)	–	[1]
2	306	760	28,479	Diverse bacterial species	0.4	[2]

Prophage identification and gene annotation were carried out using the automated PHASTER URL API (application program interface) [3], available through `https://phaster.ca`. Through this API, accession numbers were submitted and PHASTER results were returned as zipped text files, which we processed using in-house shell scripts to extract prophage lengths, classifications ("intact", "questionable" or "incomplete") and gene annotations within each genome. The accession numbers of the bacterial genomes investigated, detailed results files (containing the number of prophages present in each bacterial genome, the type of genes present on each prophage and whether the prophage is "intact", "questionable" or "incomplete") for each genome, the shell scripts and the MATLAB script used to produce Fig 1 are available at: `https://github.com/MathBioInfo/Data`

Analytical Model (Deterministic Model)

A MATLAB script which numerically integrates the system of ordinary differential equations (Eq. 1), producing Fig 3, is available at:
`https://github.com/MathBioInfo/Analytical-Model`

Computational Model (Stochastic Model)

For each simulation figure in the main text, we present results for a population of 10,000 bacterial genomes. As seen in Fig 4, for the parameter values we illustrate, the approach to a stable long-term distribution of prophages typically occurs on a timescale of hundreds of generations. We take a conservative approach in simulating "long-term" outcomes, evolving the population for 20,000 discrete bacterial generations; in each case, we also confirm by a visual comparison of the outcomes at 10,000 generations and 20,000 generations that a stable outcome has been reached.

The code for these agent-based simulations was developed in C++ and is available at: `https://github.com/MathBioInfo/Computational-Model`

References

1. Bobay LM, Touchon M, Rocha EPC. Pervasive domestication of defective prophages by bacteria. Proc Natl Acad Sci USA. 2014;111(33):12127–12132. doi:10.1073/pnas.1405336111.

2. Leplae R, Lima-Mendez G, Toussaint A. ACLAME: A CLAssification of Mobile genetic Elements, update 2010. Nucleic Acids Research. 2010;38(suppl_1):D57–D61. doi:10.1093/nar/gkp938.

3. Arndt D, Grant JR, Marcu A, Sajed T, Pon A, Liang Y, et al. PHASTER: a better, faster version of the PHAST phage search tool. Nucleic Acids Research. 2016;44(W1):W16–W21. doi:10.1093/nar/gkw387.
