# Supplementary material for: Evolution along the parasitism-mutualism continuum determines the genetic repertoire of prophages
Source: PLoS Comput Biol. 2020 Dec 4;16(12):e1008482. doi: 10.1371/journal.pcbi.1008482 (PMC7744054; doi:10.1371/journal.pcbi.1008482)
Supplement: S2 Appendix — (PDF) [file pcbi.1008482.s002.pdf]

Evolution along the parasitism-mutualism continuum determines the genetic repertoire of prophages

Amjad Khan[1], Alita R. Burmeister[2, 3], Lindi M. Wahl[1,*]

1 Department of Applied Mathematics, Western University, London, Ontario, Canada.
2 Department of Ecology and Evolution, Yale University, New Haven, Connecticut, USA.
3 BEACON Center for the Study of Evolution in Action, East Lansing, Michigan, USA.

* lwahl@uwo.ca

S2 Appendix. Fixed point and stability analysis of system of ordinary differential equations (Eq. 1)

The system of ordinary differential equations (Eq. 1) has six equilibrium points, four of which are biologically meaningful (non-negative). We will use the notation $E_i = \left(\bar{P}_{111}, \bar{P}_{011}, \bar{P}_{101}, \bar{P}_{110}, \bar{P}_{001}, \bar{P}_{010}, \bar{P}_{100}, \bar{P}_{000}\right)$, where $\bar{P}_{ber}$ denotes the equilibrium value of $P_{ber}(t)$, and describe these equilibria below.

1) The fixed point $E_0 = (0,0,0,0,0,0,0,1)$ corresponds to the complete elimination of prophages from bacterial genomes. This fixed point always exists. The eigenvalues of the corresponding linearized Jacobian are: 0, $-r_D$, $r_S - r_D$, $r_S - 2r_D$, $-r_D - r_I$, $r_S - 2r_D - r_I$, $r_L - 2r_D - r_I$, and $r_L + r_S - 3r_D - r_I$. Under the conditions $r_S < r_D$ and $r_L < 2\,r_D + r_I$, all of these eigenvalues are negative except the zero eigenvalue. Although we are unable to prove stability, extensive numerical exploration suggests that this fixed point is typically stable when $r_S < r_D$ and $r_L < 2\,r_D + r_I$.

2) The fixed point $E_B = (0,0,0,0,0,0,\frac{r_S - r_D}{r_S}, \frac{r_D}{r_S})$, corresponds to the existence of beneficial prophage genes only. This fixed point exists only if $r_S > r_D$. The eigenvalues of the corresponding Jacobian matrix are: $-r_D$, $-r_S$, $r_D - r_S$, $r_D - r_S$, $-r_I - r_S$, $-r_D - r_I$, $r_L - 2r_D - r_I$, and $r_L - r_D - r_I - r_S$. Thus the conditions for stability are $r_S > r_D$ and $r_L < 2\,r_D + r_I$.

3) The fixed point $E_{LI} = \left(0, \frac{\alpha\gamma}{r_L \eta}, 0, 0, \frac{r_D \alpha\gamma}{r_L \eta^2}, \frac{r_D \gamma}{r_L \eta}, 0, \frac{r_D^2 \xi}{r_L \eta^2}\right)$, where $\alpha = r_L - r_D$, $\gamma = r_L - 2r_D - r_I$, $\eta = r_L - r_D - r_I$ and $\xi = 2r_L - 2r_D - r_I$. E_{LI} corresponds to the coexistence of lysis and infectious genes, and exists if $r_L > 2r_D + r_I$. Eigenvalues of the corresponding linearized Jacobian are: $r_S - r_D$, $r_S - r_L$, $r_D - r_L$, $r_I + r_S - r_L$, $r_I + r_D - r_L$, $r_S + r_D + r_I - r_L$, $r_I + 2\,r_D - r_L$, and $r_I + 2\,r_D - r_L$. These eigenvalues are all negative under the two conditions $r_L > 2r_D + r_I$ and $r_S < r_D$.

4) $E_A = \left(\frac{\alpha\beta\gamma}{r_L r_S \eta}, \frac{r_D \alpha\gamma}{r_L r_S \eta}, \frac{r_D \alpha\beta\gamma}{r_L r_S \eta^2}, \frac{r_D \beta\gamma}{r_L r_S \eta} \frac{r_D^2 \alpha\gamma}{r_L r_S \eta^2}, \frac{r_D^2 \gamma}{r_L r_S \eta}, \frac{r_D^2 \beta\xi}{r_L r_S \eta^2}, \frac{r_D^2 \beta\gamma}{r_L r_S \eta^2}, \frac{r_D^3 \xi}{r_L r_S \eta^2}\right)$, where $\beta = r_S - r_D$. The eigenvalues of the Jacobian are: $r_D - r_S$, $r_D - r_L$, $2\,r_D - r_L - r_S$, $r_I + 2\,r_D - r_L$, $r_D + r_I - r_L$, $r_I + 2\,r_D - r_L - r_S$, $r_I + 3\,r_D - r_L - r_S$, and $r_I + 3\,r_D - r_L - r_S$. These eigenvalues are all negative under the conditions $r_S > r_D$ and $r_L > 2r_D + r_I$.
