# Supplementary material for: Evolution along the parasitism-mutualism continuum determines the genetic repertoire of prophages
Source: PLoS Comput Biol. 2020 Dec 4;16(12):e1008482. doi: 10.1371/journal.pcbi.1008482 (PMC7744054; doi:10.1371/journal.pcbi.1008482)
Supplement: S3 Appendix — (PDF) [file pcbi.1008482.s003.pdf]

Evolution along the parasitism-mutualism continuum determines the genetic repertoire of prophages

Amjad Khan[1], Alita R. Burmeister[2, 3], Lindi M. Wahl[1,*]

1 Department of Applied Mathematics, Western University, London, Ontario, Canada.
2 Department of Ecology and Evolution, Yale University, New Haven, Connecticut, USA.
3 BEACON Center for the Study of Evolution in Action, East Lansing, Michigan, USA.

* lwahl@uwo.ca

S3 Appendix. Transposase enrichment in incomplete prophages.

As described in the main text, we simulated the prophage population with parameter values $r_S = 1.5$, $r_D = 0.05$, $r_L = 1.5$, $r_T = 0.002$ for 20,000 generations to compare the gene content of intact and incomplete prophages. Using a strict definition for “intact” prophages, that is, only prophages containing all the genes required for excision and re-infection were considered intact, transposase genes were enriched nearly 105-fold in incomplete prophages (see Figs 1, (A) and (B)). When the classification of “intact” prophages was relaxed to prophages that contain 90% or more of the possible prophage genes, the results showed a 10.8-fold increase in transposase genes (see Figs 1, (C) and (D)).

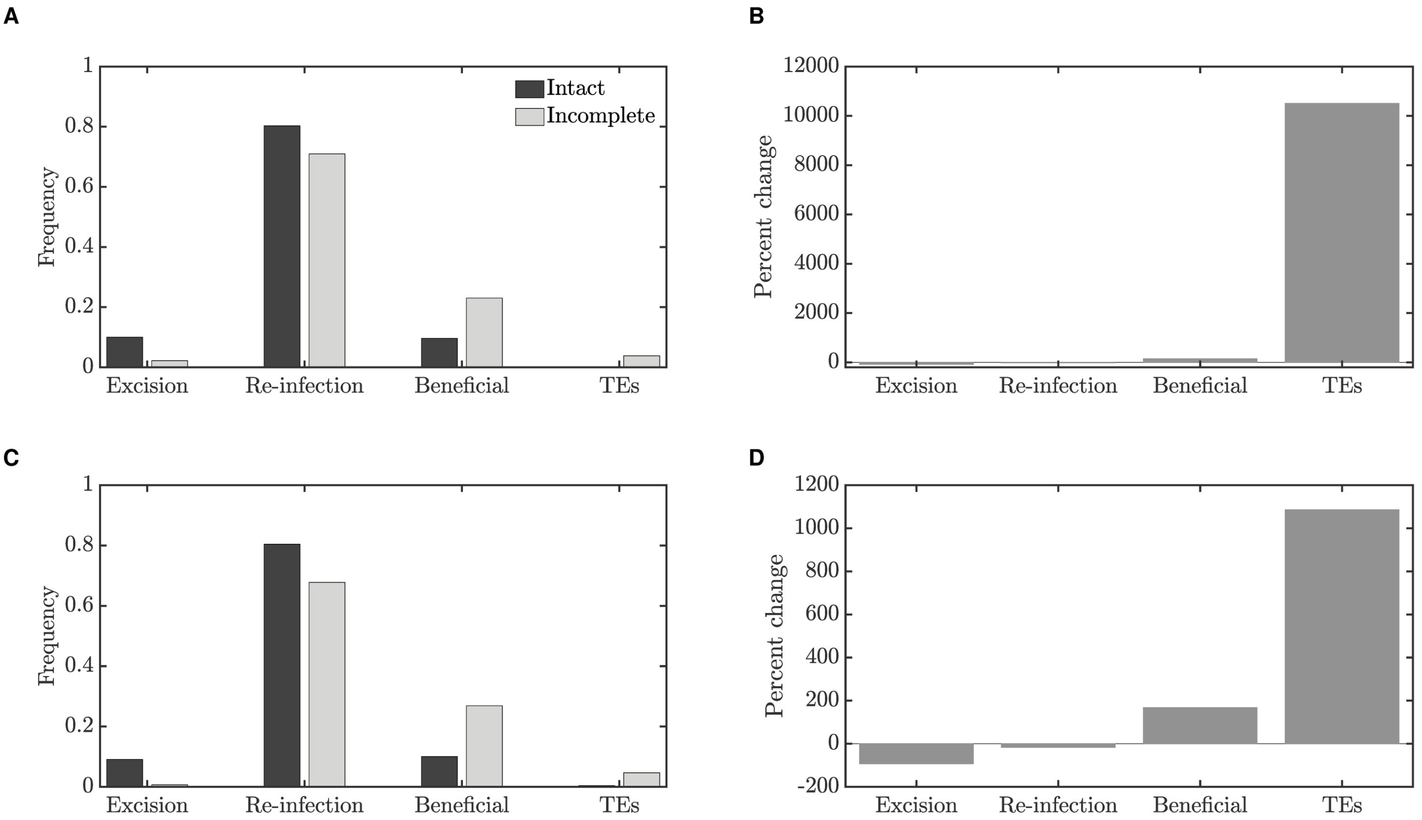

Fig 1. Gene frequencies in intact and incomplete prophages, when TEs are included ($r_S = 1.5$, $r_L = 1.5$, $r_D = 0.05$, $r_T = 0.002$). (A) and (C) Frequency of genes of each type in intact and incomplete prophages, for the computational model simulated at the persistence long-term outcome with TE disruptions; (B) and (D) Percent change in gene frequency from intact to incomplete. For (A) and (B), intact prophages are defined as sequences containing all the genes required for excision and reinfection; for (C) and (D) intact prophages are defined as sequences containing 90% or more of the possible prophage genes.
