# Supplementary material for: Evolution along the parasitism-mutualism continuum determines the genetic repertoire of prophages
Source: PLoS Comput Biol. 2020 Dec 4;16(12):e1008482. doi: 10.1371/journal.pcbi.1008482 (PMC7744054; doi:10.1371/journal.pcbi.1008482)
Supplement: S4 Appendix — (PDF) [file pcbi.1008482.s004.pdf]

# Evolution along the parasitism-mutualism continuum determines the genetic repertoire of prophages

Amjad Khan[1], Alita R. Burmeister[2, 3], Lindi M. Wahl[1,*]

**1** Department of Applied Mathematics, Western University, London, Ontario, Canada.
**2** Department of Ecology and Evolution, Yale University, New Haven, Connecticut, USA.
**3** BEACON Center for the Study of Evolution in Action, East Lansing, Michigan, USA.

* lwahl@uwo.ca

## S4 Appendix. Transposase distribution.

Fig 1 shows histograms of the total number of transposase sequences identified in each prophage in the two datasets.

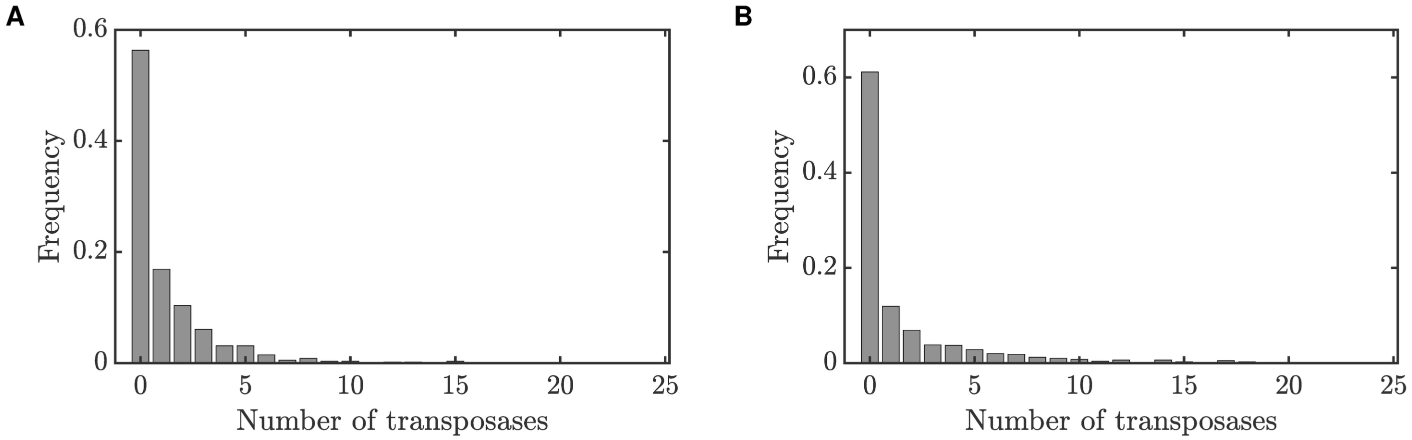


**Fig 1. Frequency histogram of the number of transposase sequences identified in each prophage in** (A) Data Set 1; (B) Data Set 2.
